# Exercise physiologists use of pain neuroscience education for treating knee osteoarthritis: A qualitative interview study

**DOI:** 10.1002/msc.1631

**Published:** 2022-03-17

**Authors:** Adrian Ram, John Booth, Jeanette M. Thom, Matthew D. Jones

**Affiliations:** ^1^ Faculty of Medicine & Health School of Health Sciences The University of New South Wales Sydney New South Wales Australia; ^2^ Centre for Pain IMPACT Neuroscience Research Australia Sydney New South Wales Australia

**Keywords:** exercise therapy, OA practice guidelines, osteoarthritis, pain neuroscience

## Abstract

**Objectives:**

To explore how Australian exercise physiologists (EPs) utilise pain neuroscience education (PNE) in their management of patients with knee osteoarthritis.

**Methods:**

A semi‐structured interview concerning a knee osteoarthritis vignette was designed to understand each participant's beliefs about physical activity, pain, injury and coping strategies and quantify their use of pain neuroscience concepts. Themes were derived from pre‐determined pain target concepts as well as others that emerged from thematic analysis.

**Results:**

Thirty EPs (57% male, mean clinical experience 7 years (SD 7.1) participated in the semi‐structured interviews. 13 themes emerged. EPs primarily focussed on: (1) active treatment strategies are better than passive, (2) pain and tissue damage rarely relate, and (3) learning about pain can help individuals and society. Other themes included the use of biomedical‐based education, pain during exercise and delivery of PNE. Underutilised themes included the role of the brain in pain, validation that pain is real and personal, the concept of danger sensors as opposed to pain sensors, and pain depends on the balance between safety and danger.

**Conclusion:**

EPs primarily advised on active treatment approaches (e.g. exercise and self‐management). Quality of care is likely to improve through increasing focus on the systemic benefits of exercise in overcoming psychological barriers (e.g. fear avoidance and pain catastrophising) that may prevent exercise treatment engagement. Broadening PNE to reconceptualise knee osteoarthritis pain as a sign of an overprotective nervous system, rather than structural damage, may facilitate greater patient engagement in exercise therapies, thus improving patient outcomes.

## 1 INTRODUCTION

Osteoarthritis (OA), a progressive joint disease, is a leading cause of pain and disability worldwide (AIHW, 2019). Knee OA is characterised by pain, stiffness and swelling, resulting in functional limitations and reduced life quality. Clinical practice guidelines recommend conservative management, including education, exercise, drug therapy, and weight management (Bannuru et al., 2019; Bierma‐Zeinstra et al., 2020). A contemporary understanding of OA recognises knee health to be influenced by the interaction of biopsychosocial factors that influence inflammatory processes and sensitisation in the knee (Caneiro et al., 2020). Hence, health professionals must ensure they deliver patient‐centred care in line with guidelines that address the multitude of factors contributing to the patient's knee health and pain experience (Bunzli et al., 2019; Caneiro et al., 2020).

Education is consistently recommended in clinical care guidelines for knee OA (Bannuru et al., 2019; Lin et al., 2020). Improving patients' understanding of knee OA and implementing positive behaviour change are key underlying concepts of education interventions. This includes reconceptualising knee OA pain as a modifiable symptom related to the interplay of biopsychosocial factors rather than merely structural changes at the knee joint (Caneiro et al., 2020). Education should help inform patients how contextual factors can influence their pain and disability (e.g. comorbidities, catastrophising, fear avoidance, unhelpful beliefs, and pain sensitisation (Fingleton et al., 2015; French et al., 2015). Misinformed beliefs and misconceptions about knee OA can poorly influence patient outcomes relating to activity engagement and healthy behaviours (Darlow et al., 2018). Therefore, clinical practice guidelines recommend using positive behavioural change strategies and education to challenge unhelpful beliefs and misconceptions about knee OA (Ram et al., 2020).

There is advocacy at both national and international levels for clinicians to incorporate pain education into chronic pain treatments (Fishman et al., 2013; International Pain Summit of the International Association for the Study of Pain, 2011). Pain neuroscience education (PNE) is one educational strategy used to teach patients about the neurobiology of pain (Moseley & Butler, 2015). As a standalone therapy, PNE does not produce clinically meaningful changes in pain and disability (Louw et al., 2020). However, PNE delivered with active treatment modalities, including exercise (Moseley & Butler, 2015), can evoke positive changes including reduced kinesiophobia and pain catastrophising—both common in OA (Darlow et al., 2018; Somers et al., 2009). While current clinical guidelines for knee OA recommend patient education, they do not include specific recommendations for PNE.

Accredited exercise physiologists (EPs) are allied health professionals who commonly treat knee OA. A recent survey study found that in relation to knee OA guideline recommendations, only 61% of EPs treating knee OA provided education on self‐management principles, 56% on weight management and 51% on pain management, including PNE (Ram et al., 2020). In this study, the authors did not assess the quantity or quality of PNE delivered by EPs. Given the potential benefits to musculoskeletal pain from treatments that combine PNE and exercise, clarification of the PNE component of EP interventions for knee OA is warranted.

This study aimed at determining (1) the content of PNE interventions delivered by EPs to patients with knee OA and (2) how closely these PNE interventions align with target pain science concepts.

## 2 METHOD

### 2.1 Design

A qualitative semi‐structured interview was used to examine EPs' delivery of key pain neuroscience concepts during an education session based on a hypothetical patient with knee OA. Semi‐structured interviews were conducted between August 2019 and July 2020. Ethics approval was obtained from the institutional Human Research Ethics Committee (#HC190038). The guidelines for good reporting of a qualitative study (COREQ) were followed (Tong et al., 2007).

### 2.2 Participants

Participants were recruited via expression of interest from a previous study (Ram et al., 2020) and by online advertising across various social media platforms and professional network groups (e.g., State Chapters of Exercise and Sports Science Australia (the EP accrediting body in Australia)). EPs were sampled from multiple practices across Australia, including both private and public sectors. All participants provided informed consent before beginning the interview.

#### 2.2.1 Sample size calculation

Sample size calculations for thematic analysis were used (Marshall et al., 2013). We sought to identify at least 5 instances of the least prevalent theme in a population where the theme prevalence was estimated to be 30%. On this basis, 25 participants were required to detect this with 90% power. This sample size is comparable to previous similar studies (Holden et al., 2009; MacKay et al., 2018; Teo et al., 2020) and is in line with the recommended number of interviews for qualitative research (i.e., 20–30; Vasileiou et al., 2018). Thus, we aimed at recruiting 30 participants to capture a broader sample of EPs, with 15 from metropolitan and 15 from regional areas, including a range of ages, years of clinical experience, and representation of genders.

### 2.3 Intervention

Participants who expressed interest were contacted via phone or email, and an invitation containing study information was sent. One week after initial contact, participants were followed up to organise a meeting time. Individuals who did not respond to the initial contact or responded but did not wish to participate were not contacted again. One day before the interview, participants were sent a knee OA patient vignette, including the questions to be asked during the interview (supporting information S1). Two researchers (AR and JB) piloted the interview questions with other EPs (*n* = 3) who regularly treat people with musculoskeletal injuries to assist in the development of the interview schedule. Participants who completed the interview were entered into a draw to win 1 of 10 $50 gift vouchers.

### 2.4 Interview protocol

One researcher (AR; a male EP with clinical experience in musculoskeletal rehabilitation and trained in pain management) conducted all the semi‐structured interviews, either face‐to‐face or online via video conference (Zoom™ platform). Before commencing the interview, participants were debriefed on the study's purpose, and consent was gained for the interviews to be recorded. Each interview lasted approximately 20–30 min. The interview was guided by 10 questions formulated by the primary research aim (supporting information S2). These questions were targeted to understand the education each EP would provide in their treatment approach and to understand each clinician's beliefs about physical activity, pain, injury and coping strategies with reference to the knee OA vignette. They were also designed to understand the PNE concepts each EP would provide in a similar clinical situation. All questions were linked to the patient's vignette with knee OA, which included descriptions about history, symptoms, imaging findings, and relevant psychosocial factors. Upon completing the interview, participants were asked about their years of clinical experience and whether they had received any formal training in PNE. All interviews were conducted in privacy between the researcher and participant.

### 2.5 Data analysis

All interviews were digitally recorded and professionally transcribed verbatim (transcribeme.com). Data sampling continued until the sample size was reached. Interviews were not repeated on completion, and transcripts were not returned to participants for comments or corrections. Themes were identified using a qualitative content analysis approach (Elo & Kyngäs, 2008) with deductive codes constructed from pre‐determined PNE target concepts as outlined by Moseley and Butler (2015). These target concepts are commonly used in clinical practice and provided the benchmark for decisions concerning the nature of PNE delivered by EPs. Other themes that emerged that were not related to these pre‐defined PNE concepts were also coded and analysed using an inductive approach. Inductive coding (Braun & Clarke, 2006) was sampled on the initial 10 transcripts by one researcher (AR), using NVivo (v12.0, QSR International). A second researcher (MJ) blind to this initial coding re‐coded the first three transcripts, and the research team (MJ, JB, JT and AR) met to compare codes. In these meetings, the research team further redefined the coding using an iterative process in which nodes were added, removed, grouped and redefined (Song et al., 2019).

## 3 RESULTS

### 3.1 Participants

A total of 47 EPs were recruited, of which 30 (64%) completed the semi‐structured interview. The participant characteristics are outlined in Table 1. Of those who did not complete the study, 13 (28%) did not respond to the mail out invitations, and 4 (8%) chose not to participate. For completers, 13 (43%) were recruited via expression of interest from an earlier survey study (Ram et al., 2020), and the remaining 17 (57%) via social media. Participants were predominantly male (57%, *n* = 17), practising in New South Wales (53%, *n* = 16), with varied clinical experience (range 1–28 years; median = 7 years, interquartile range 7.25 years).

**TABLE 1:** Demographic data of the accredited exercise physiologists

Variable	Category	Sample (*n* = 30) *N* (%)
Sex	Male	17 (57)
	Female	13 (43)
Clinician experience	0–5 years	18 (60)
	6–10 years	7 (23)
	>10 years	5 (17)
State/territory	New South Wales	16 (53)
	Queensland	5 (17)
	South Australia	3 (10)
	Western Australia	3 (10)
	Victoria	2 (7)
	Tasmania	1 (3)
Practice location	Rural	3 (10)
	City/Metropolitan	27 (90)

### 3.2 Thematic analysis

Analysis resulted in 13 themes, including 10 pre‐determined PNE target concepts and three additional themes. No new themes emerged in the remaining 20 transcripts than the initial 10 that were piloted for coding and thematic analysis, indicating data saturation had been reached (Guest et al., 2006). Themes are summarised below with relevant examples to support each theme provided in Table 2.

**TABLE 2:** Summary of the PNE themes and other emerging themes with supporting quotes by the EP participants to the knee OA vignette provided in ranked order of responses

	Themes	EP quote	
PNE 1	Active treatment strategies are better than passive (*n* = 30, 100%)	‘activity pacing to help with improving activity and that it's okay to exercise whilst you have symptoms, other strategies to help with sort of winding down pain symptoms as well, so not just physical activity but perhaps other stress management techniques, mindfulness, other relaxation, breathing, thought management approaches’ (EP#3).	
		‘I would also tell her a bit more about how exercise actually does work. What the function of it is. How the muscles support the joints. And how the joint releases synovial fluid to help lubricate the knee…And then how body alignment, posture, and regular function can actually help reduce inflammation and stress, which helps reduce the pain’ (EP#10).	
		‘Movement is healing. It's really important for you to move the joint to start the healing process.’ And that's not like big walks or anything. That could just be simply daily joint mobility exercises to keep the fluid flushing the joint out,’ (EP#19)	
		‘You're not damaging the joint when you exercise’ (EP#12)	
PNE 2	Learning about pain can help the individual and society (*n* = 30, 100%)	‘I think pain education might help her with her self‐efficacy, which will help her feel better about movement and daily activities’ (EP#1)	
		‘I think pain education could be helpful to try and give her a bit more of an active coping mechanism like exercise and reduce her fear associated with exercise.’ (EP#17)	
		So I guess it would be to look at improving her self‐efficacy, feelings of control around what control she has. I think a better understanding of the disease can help with catastrophising pain. And when you get rid of a lot of the unknowns then people can deal with it a lot better if they understand it better.’ (EP#25)	
		‘Perhaps I would direct them to some self‐learning about pain education as well. I might actually tell them to go watch a video or read some articles depending on how interested she is’ (EP#26)	
PNE 3	Pain and tissue damage rarely related (*n* = 26, 87%)	‘the overreaching thing with pain education… is that pain does not equal damage. Pain does not mean you’re causing yourself damage…pain is just a warning sign that our body produces.’ (EP#5)	
		‘there’s multiple contributors to her experience, You could draw on actually, well, that’s just another sign that your whole sort of system has become a bit overprotective, as opposed to that being a sign of more tissue damage, so I wouldn't necessarily be concerned.’ (EP#4)	
		I would probably reassure her that the crepitus sounds, the clicks and grinding noises, that’s quite normal. And it’s not necessarily an indication of damage or danger. And lots of people experience that clicking and cracking in their joints when they’re not experiencing pain. (EP#29)	
Other	Metaphors and analogies (*n* = 22, 73%)	Positive‐ Safety Messages	Negative‐ Danger Messages
		‘Pain is Like a House Alarm…over time, that house alarm can get more sensitive, so it might go off when the wind blows…So exercise is trying to desensitise that whole alarm system, so exercise isn't so uncomfortable.’ (EP#17)	‘No, no. Not the crepitus. The bone that's sort of rubbing on each other, the wear and tear’ (EP#14)
		‘one that I like to use is your body shows wrinkles over time.’ (EP#10)	‘she has worn away things like the cartilage and the meniscus, then obviously, we're going start to get a bit of bone‐on‐bone.’ (EP#16)
		‘Movement is healing.’ (EP #19)	
		‘Hurt doesn’t mean harm,’ (EP#12)	
Other	Biomedical based education (*n* = 22, 73%)	‘There might be a movement compensation. So if the knee's sore, then you'd shift the weight to the left hip, which would then make that worse, which would then shift the body… by looking at her whole function…we might look at the gluteal muscles for stability. We might look at the leg, knee muscles for strength and balance…we educate her on correcting techniques and correcting walking’ (EP#10)	
		‘If it's potentially in a movement that she hasn't built her tolerance up in, so just kind of reassure her that at the moment, you're avoiding this activity because it is causing pain, but you haven't had enough time to work on your stability and strength to build up that tissue tolerance so that it is a non‐painful movement. It's a lot of reassurance.’ (EP#13)	
		‘biomechanically, other muscle groups are going to be having to help out to walk the certain way that you’re currently walking to nurture that side. So it may cause other muscles to become overtight and stiff, and that’s hence why you may be getting extra pain in those areas.’ (EP#28)	
PNE 4	Pain relies on context (*n* = 20, 67%)	‘Looking at other factors in her life. If she's particularly stressed, we know that that can make any level of pain get turned up quite a bit. So seeing if there's other factors in her life that are causing her to be more susceptible to pain. We'd have to try and dig into that a bit more.’ (EP#16)	
		‘there's multiple contributors to her experience, you might pull out things like her stress or maybe some of these thoughts and beliefs or other sort of lifestyle factors which might be sort of unhelpful or sort of contributing to her general sort of level of sensitivity. You could draw on actually, well, that's just another sign that your whole sort of system has become a bit overprotective, as opposed to that being a sign of more tissue damage, so I wouldn't necessarily be concerned.’ (EP#4)	
		‘a little bit of information talking about stress, which she feels like her symptoms might increase when she has an increase in stress, so just reviewing those other inputs…there's a bit more information about her stress, her sleep, her mood, social support, workplace support, even on environmental, like accessibility to physical activity spaces and all that sort of stuff, just to sort of see whether those other concepts need a little bit more attention in an individualised education approach.’ (EP#4)	
Other	Exercise and pain (*n* = 20, 67%)	‘If the pain stays the same or even reduces, then to keep exercising. But if we see an increase in pain, then to stop’ (EP#16)	
		‘I like to give them a traffic‐light system…green is if you start doing a movement and it starts to feel better after a couple of times, then you continue on. It's very safe…orange light refers to if it's uncomfortable, and it remains uncomfortable throughout the movement ‐ it doesn't really get drastically worse or any better ‐ it's also safe to continue…if it's a red light, if it's drastically getting worse, and every sort of rep is becoming more and more symptomatic, then we stop that movement and try something else. I always try to make sure that they understand that that movement is not evil and that doesn't mean that they can't ever do that; it's just today, the body's a bit sensitive. (EP#6)	
		‘discuss with her what is normal, what is not normal, what is acceptable pain, talking that there is a point where you start exercising and your pain can increase to a manageable level. And if it stays at that point, then that is acceptable. And as long as it goes back down when you stop exercising or soon after, that's a completely acceptable amount of pain. But if your pain starts to increase and then continues to increase throughout your exercise, then perhaps you need to modify your exercise.’ (EP#26)	
PNE 5	We are bioplastic (*n* = 15, 50%)	‘I would reassure her. I would, I guess, educate her on the bioplastic nature of tissues and show her that they can recover. I think when she does get pain, if it's potentially in a movement that she hasn't built her tolerance up in.’ (EP#13)	
		‘I would say it's not an indication that she's causing further damage. It's more an indication that she's created these pain pathways now and reinforced them psychologically so that she thinks potentially, every time she's moving her knee, in her head, she thinks it's pain. And she thinks that it's getting worse… she has reinforced a lot of those neural pathways.’ (EP#19)	
PNE 6	Pain is one of many protective outputs (*n* = 10, 33%)	‘pain is like a fire alarm or something that is designed to protect you…and emphasise how alarms can be oversensitive. So sometimes a fire alarm goes off for a burnt piece of toast, or the car alarm goes off when someone just touch your back windows, so try to describe that sort of oversensitive alarm.’ (EP#4)	
		‘the fact that she's feeling pain in other joints or feeling symptoms in other joints is evidence of a possible turning up of the volume of the central nervous system. So I'd talk about the Explain Pain stuff about pain being protective and the brain interpreting what's going on with her as evidence that it needs to create pain to protect her.’ (EP#12)	
PNE 7	Pain involves disturbed brain activity (*n* = 9, 30%)	‘First of all, we go back to the basic principles of pain education, that pain is a 100% construct of the brain, and just trying to deliver that in a nice manner, try and ease it to her softly that it's not that she's making things up, it's that its pain is real, but pain is a 100% construct of the brain,’ (EP#26)	
		‘I then let them know that the pain is created by the brain 100% of the time but that there's many influencing information that goes into the brain for it to make that decision of producing pain.’ (EP#13)	
PNE 8	Pain is normal, personal and real (*n* = 8, 27%)	‘I always reassure the patient that their pain is real.’ (EP#13)	
		‘Similar to the nail in the shoe. There's a story about a worker who had a nail gun. He was doing his work and put a nail through the middle of this boot. He was in the world's worst pain. They rushed him off to the hospital. They cut the boot off, and they found that the nail went between the big toe and the second toe. And all of a sudden, his pain disappeared. He was in real pain, but he had no damage.’ (EP#26)	
PNE 9	Pain depends on the balance of danger and safety (*n* = 7, 23%)	‘So pain in the body is something…created by the brain as a warning system if it perceives more threat than safety or more danger than safety, it'll send basically a warning signal which is what the pain often is so that it's not necessarily causing more damage, or it's not necessarily harming the joint even though it might hurt, and it's often not necessarily due to the arthritis either.’ (EP#24)	
		‘I wouldn't be prescribing this exercise if I didn't think it was safe for you. And I guess just promoting this idea of, ‘You're safe to move. Even if you have a flareup, it doesn't mean you've damaged your joint. It's not fun, but it's safe.’ (EP#29)	
PNE 10	There are danger sensors, not pain sensors (*n* = 5, 17%)	‘Explaining pain…that it is a warning system…so for instance, her knee isn't deciding she's in pain. It sends a message of potential damage, or it sends a message of warning to the brain, and the brain then responds with the perception of pain.’ (EP#5)	
		‘the clients may feel that there is damage being done, but sometimes the pain is useful, overall, in terms of when learning what things are bad and what things to avoid, but sometimes it can be overprotective and over time, if it becomes too protective and the warning signals or danger signals are too heightened, it can be a bit unrealistic in terms of what the client can actually do and can't do.’ (EP#28)	

Abbreviations: EP, exercise physiologist, OA, osteoarthritis; PNE, pain neuroscience education.

### 3.3 10 PNE themes

#### 3.3.1 Active treatment strategies are better than passive

All EPs (*n* = 30) provided education on the importance of active, as opposed to passive, treatment strategies. Respondents focussed on teaching patients about the benefits of exercise (93%, *n* = 28), general activity and movement (77%, *n* = 23) and discussion/education on the benefits of active coping strategies (73%, *n* = 22). Respondents stated they would educate patients to engage more actively in their pain rehabilitation. A minority of EPs delivered education regarding interdisciplinary treatment with other allied health professionals (e.g., psychologist, physiotherapist; 13%, *n* = 4), psychological strategies (e.g., mindfulness; 13%, *n* = 4), diet (10%, *n* = 3) and self‐massage (10%, *n* = 3). Few EPs (7%, *n* = 2) promoted passive coping strategies.

#### 3.3.2 Learning about pain can help the individual and society

All EPs believed it was important to teach about pain (*n* = 30). Educational content regarding the benefits of PNE varied in nature, with most respondents believing PNE would help improve patient self‐management and coping strategies (87%, *n* = 26), including engagement in exercise (53%, *n* = 16). EPs also used PNE to address patient psychosocial factors, including stress, anxiety, and fear avoidance (47%, *n* = 14), and some EPs provided a rationale for how these psychosocial factors influence knee OA pain (33%, *n* = 10).

#### 3.3.3 Pain and tissue damage rarely relate

A popular PNE target concept discussed by EPs was the (lack of) relationship between tissue damage and pain (87%, *n* = 26). This included content explaining that pain could occur without the presence of tissue. Damage, the role of the nervous system and brain in pain, and pain as a warning signal rather than a sign of tissue damage. Advice also included reassurance about clicking joints (crepitus) not being a sign of tissue damage or degeneration but a normal part of ageing (27%, *n* = 8). General advice from EPs was to remain active despite pain, and that exercise/physical activity is safe and will not cause further damage.

#### 3.3.4 Pain relies on context

Education detailing how patient context influences pain was a common theme across the interviews (67%, *n* = 20). Education was broad in content and included advice on how pain can be negatively influenced by unhelpful beliefs and thoughts about pain (47%, *n* = 14), mental health (e.g., anxiety, stress; 30%, *n* = 10), activity levels (27%, *n* = 8), sleep (10%, *n* = 3) and a low support network (10%, *n* = 3).

#### 3.3.5 We are bioplastic

This theme captured EPs explaining the nature of the ‘bioplastic’ nervous system, which becomes highly sensitive/protective (e.g., central sensitisation) when pain persists (50%, *n* = 15). Education on treatment strategies to help desensitise the nervous system included explaining the benefits of exercise treatment (e.g., activity pacing; 37%, *n* = 11), reassurance (23%, *n* = 7) and psychological approaches to pain management (7%, *n* = 2).

#### 3.3.6 Pain is one of many protective outputs

Few respondents provided education on pain being a protective system (33%, *n* = 10). Furthermore, no respondents educated patients on other protective systems in the body (e.g., immune, endocrine, motor, and autonomic)

#### 3.3.7 Pain involves disturbed brain activity

Education on the general role of the brain in pain was explored by some EPs (27%, *n* = 9). However, specific education on the multiple brain areas involved in pain processing was not discussed by any respondents.

#### 3.3.8 Pain is normal, personal, and real

A minority of EPs identified validating pain experiences as real and an unpleasant experience of what the brain judges to be threatening. Education was minimal and included reassuring and validating a patient's pain experience as normal and reflective of their current situation (27%, *n* = 8).

#### 3.3.9 Pain depends on the balance of danger and safety

EPs counselled on how pain depends on the balance between safety and danger (23%, *n* = 7). This saw EPs introduce concepts such as ‘Danger in Me’ and ‘Safety in me’. EPs also highlighted the importance of positive, optimistic language to create a sense of safety for the patient to improve exercise engagement.

#### 3.3.10 There are danger sensors, not pain sensors

The concept of nociceptors (danger sensors) was seldom discussed (17%, *n* = 5). This theme saw EPs refer to the sensitised nervous system and pain as a protector.

### 3.4 Other themes

#### 3.4.1 Education about pain during exercise

This theme arose in the majority of EPs (67%, *n* = 20) who reported they would allow patients to experience pain during exercise as long as it did not increase with activity/exercise. Education that identified the importance of correct biomechanics and addressed the relationship between irregular movement patterns/activity and pain was reported by half the EPs (50%, *n* = 15). During exercise prescription, the most common approach used by EPs for interpreting the patient's level of pain was through the use of descriptors such as ‘tolerable’ and ‘manageable’ (53%, *n* = 16). EPs (20%, *n* = 6) reported using a quantified pain measurement (e.g., 0–10 pain scales) during exercise. Few EPs included education about delayed muscle soreness through exercise and effective pacing (23%, *n* = 7).

#### 3.4.2 Use of biomedical based education models

Education consistent with the biomedical based model (73%, *n* = 22) included advice on how anatomical and biomechanical changes in the knee result in altered movement patterns and pain. EPs emphasised poor biomechanics (70%, *n* = 21), muscle weakness (37%, *n* = 11) and the disease OA itself (37%, *n* = 11) as being contributing factors to chronic pain. Most EPs reported that additional imaging (e.g., MRI) was not required (77%, *n* = 23). Several EPs who did not oppose imaging (20%, *n* = 6) counselled patients that further imaging would not change their treatment approach. Three EPs (10%) advised further investigation and screening if no improvement was achieved after an exercise intervention.

#### 3.4.3 Metaphors and analogies

Most EPs reported using metaphors and analogies during their PNE intervention (73%, *n* = 22). This included explaining concepts such as central sensitisation, the role of the brain in pain, and how active treatment can improve patients ‘function and symptoms with OA. Few EPs (7%, *n* = 2) mentioned metaphors or analogies that reinforced danger messages such as ‘the bone…rubbing on each other, the wear and tear’ (*EP#14*). Education was targeted to the patient's presentation in most cases (57%, *n* = 17), which saw EPs propose using videos, visual illustrations, pictures and books (27%, *n* = 8).

### 3.5 PNE delivery

Knowledge, training and experience in delivering PNE interventions varied considerably between participants. EPs reported their experience in delivering PNE to be based on (1) their self‐learning of pain science concepts via scientific based‐literature, podcasts, books and lectures (67%, *n* = 20) and (2) from their daily professional practice as an EP, which included consulting with colleagues and other health professionals (60%, *n* = 18). Less than half the EPs (43%, *n* = 13) reported that they had received formal training (e.g., professional development courses, lectures, conference and pain management courses) in delivering pain science education. Few EPs (*n* = 10, 33%) reported tertiary education (e.g., university) as the primary source of PNE knowledge. In most cases, EPs stated that practising the delivery of PNE was limited or absent from their university curriculum. Several EPs (7%, *n* = 2) mentioned barriers to implementing PNE interventions, including time constraints and cultural adaptations.

## 4 DISCUSSION

To our knowledge, this is the first study to investigate the PNE delivered by Australian EPs treating a patient with knee OA. This study used interviews based on a knee OA vignette to quantify the pain target concepts (Butler & Moseley, 2017) delivered by EPs during treatment. The PNE concepts commonly addressed by EPs were ‘active treatment strategies over passive’, ‘learning about pain helps the individual and society’ and ‘pain does not equal tissue damage’. These concepts concerned shifting patients towards more active treatment strategies (e.g., exercise, graded activity and self‐management), including reassurance that pain does not always relate to tissue damage. Minimal advice was placed on the PNE themes: ‘pain involves distributed brain activity’, ‘pain is one of many protective outputs’, and ‘all pain is real’. Greater emphasis on these PNE themes could assist EPs to better reassure and validate the patient’s pain experience and reconceptualise pain from a marker of tissue damage to an output of the brain based on a perceived need to protect against potential threat (Louw et al., 2020; Moseley & Butler, 2015; Nijs et al., 2011). Improving the content and quality of PNE delivered during exercise for knee OA may increase the likelihood of positive change to unhelpful patient beliefs and behaviours. This approach would also align current EP practices more closely with the biopsychosocial model of care.

In agreement with clinical practice guidelines for knee OA, EPs strongly recommended an active treatment approach, including education, activity/exercise and self‐management (Bannuru et al., 2019). Consistent with previous research (Ram et al., 2020), EPs generally promoted resistance training and provided education on the benefits of strengthening the muscles around the knee. This is not surprising given that EPs' university training is heavily geared towards promoting exercise and behaviour change for various chronic conditions. Several EPs (∼20%) recommended unwarranted imaging, describing knee OA as a degenerative ‘wear and tear’ condition. Inappropriate use of imaging and language used to describe findings such as ‘degeneration’ is likely to shift the patient’s focus towards a biomedical model of knee OA and further strengthen the patient’s belief that pain equals tissue damage (’Caneiro et al., 2021). Patients who are misinformed about pain are also likely to consider pain as more threatening and have shown to experience lower pain tolerances, increased pain catastrophising and employ poorer coping strategies (Jackson et al., 2005). Health professionals should target maladaptive pain beliefs and behaviours (e.g., pain = damage, exercise is unsafe) through education and exercise to instil a sense of optimism and reassurance, facilitating positive treatment expectation and engagement (The Royal Australian College of General Practitioners, 2018).

Despite considerable evidence on the impact of psychosocial factors on chronic pain (Tracy, 2017), just under half the EPs reported addressing psychosocial factors (e.g., anxiety, catastrophising and fear avoidance) and only 20% advised on other evidence based treatments (e.g., psychologist, dietician). These findings coincide with another knee OA study showing a lack of advice from physiotherapists around psychological therapies and support (Teo et al., 2020). Current clinical practice guidelines highlight the importance of assessing and managing psychosocial factors in providing high quality care for musculoskeletal pain (Lin et al., 2020). EPs’ quality of care can improve through extending education to include the psychological benefits of exercise, providing education about other effective treatments, and engaging patient’s in interdisciplinary treatment, which has shown to benefit pain self‐efficacy, disability, and social re‐engagement for knee OA (Williams et al., 2020).

Effective pain treatment requires health professionals to adopt a holistic understanding of pain across several domains, including neuroscience (Louw et al., 2020), immunology, endocrinology, sociology, psychology, and philosophy (Thacker & Moseley, 2012). Based on our results, most EPs acknowledged PNE as an essential aspect in promoting self‐management, yet provided little education on PNE target concepts such as pain and context, the bioplastic nervous system, pain and other protective systems, and the brain’s role in pain and nociception. The limited range of PNE target concepts delivered may be due to EPs’ not understanding pain neuroscience and neurobiology at a depth required to provide education about these concepts (Ram et al., 2020). Most EPs (87%) understood that pain and tissue damage poorly relate, yet 73% of EPs continued to discuss the significance of biomechanical/biological contributors to pain. A bias towards more biomedical education and treatment approaches has also been shown in other health professionals (e.g., physiotherapists) treating knee OA (Teo et al., 2020). Considering OA is a condition characterised by heightened pain sensitivity and inflammation (’Mills et al., 2019), explaining changes in terms of a more sensitive nervous system that becomes better at producing pain, as opposed to further structural damage (Caneiro et al., 2020), would be a primary education target to reduce threat and promote engagement in exercise (Nijs et al., 2011).

Clinicians with negative beliefs about pain are likely to emphasise/advise on unhelpful behaviours, thus potentially reinforcing unhelpful coping strategies (Darlow et al., 2012). In our study, most EPs reported their university training did not encompass adequate pain education curriculum, attributing their learning to colleagues and subsequent professional development. Poor undergraduate pain curricular is also evident across other health professionals (Alves et al., 2013; Shipton et al., 2018). This highlights a need for standardised pain curricular content in entry level undergraduate courses (Louw et al., 2020), including interprofessional development for already practising health professionals. Targeting education about the psychosocial and contextual factors of each patient is more likely to evoke positive changes in patient beliefs and behaviour for clinicians with an in depth knowledge and understanding of contemporary pain science (Caneiro et al., 2021). With additional training, EPs could impart a more holistic approach and educate patients about the role of contextual factors on pain, thus improving pain self‐efficacy, pain catastrophising and kinesiophobia to assist engagement in exercise.

There were several limitations to our study. As participants were recruited from both social media and professional networks, our data may show selection bias towards those who actively use these networks. Most EPs (60%) were relatively new to clinical practice, which may have underrepresented the current professional knowledge in PNE. Furthermore, EPs may not have responded as they usually would in a clinical environment. Attempts were made by reassuring participants that all views would be respected. However, is it possible that the quality of education provided may have been over‐represented compared to a real‐world setting. Finally, during the interview, the nature of the questions may have steered EPs towards some answers (target pain concepts) but not others. However, pilot testing (*n* = 3) revealed that the questions did provide EPs with the opportunity to address all 10 target PNE concepts.

In conclusion, EPs delivered education on active treatment approaches for knee OA. Reassurance on the relationship between pain and tissue damage was a primary pain concept discussed by EPs. Quality of care may be improved by increasing focus on the psychological aspects of pain and by improving clinician knowledge on modern pain science principles. This would lead to treatment provision that aligns more closely with a biopsychosocial model of care may help guide positive health behaviours, improved self‐management and engagement in exercise therapy for patients with knee OA.

## ETHICS STATEMENT

Ethics approval was obtained from the institutional Human Research Ethics Committee (project number HREC #HC190038).

## AUTHOR CONTRIBUTIONS

Matthew D. Jones, John Booth and Jeanette M. Thom conceived and designed the research; Adrian Ram completed the interviews; Matthew D. Jones and Adrian Ram analysed the data and interpreted the results; Adrian Ram drafted the manuscript; Adrian Ram, Matthew D. Jones, John Booth and Jeanette M. Thom edited and revised the manuscript; Adrian Ram, Matthew D. Jones, John Booth and Jeanette M. Thom approved the final version of the manuscript.

## Supporting information

Supporting Information S1

Supporting Information S2

## Data Availability

The data that support the findings of this study are available from the corresponding author upon reasonable request.

## References

[msc1631-bib-0001] AIHW . (2019). Australian Burden of Disease Study: Impact and causes of illness and death in Australia 2015. p. 220.

[msc1631-bib-0002] Alves, R. D. C. , Tavares, J. P. , Funes, R. A. C. , Gasparetto, G. A. R. , Silva, K. C. C. D. , & Ueda, T. K. (2013). Análise do conhecimento sobre dor pelos acadêmicos do curso de Fisioterapia em centro universitário. Revista Dor, 14, 272–279. http://www.scielo.br/scielo.php?script=sci_arttext&pid=S1806‐00132013000400008&nrm=iso

[msc1631-bib-0003] Bannuru, R. R ., Osani, M. C ., Vaysbrot, E. E ., Arden, N. K ., Bennell, K ., Bierma‐Zeinstra, S. M. A ., & McAlindon, T. E . (2019). OARSI guidelines for the non‐surgical management of knee, hip, and polyarticular osteoarthritis. Osteoarthritis and Cartilage, 27(11), 1578–1589. 10.1016/j.joca.2019.06.011 31278997

[msc1631-bib-0004] Bierma‐Zeinstra, S. , van Middelkoop, M. , Runhaar, J. , & Schiphof, D. (2020). Nonpharmacological and nonsurgical approaches in OA. Best Practice & Research Clinical Rheumatology, 34(2). 101564. 10.1016/j.berh.2020.101564 32773214

[msc1631-bib-0005] Braun, V. , & Clarke, V. (2006). Using thematic analysis in psychology. Qualitative Research in Psychology, 3(2), 77–101. 10.1191/1478088706qp063oa

[msc1631-bib-0006] Bunzli, S. , O’Brien, P. , Ayton, D. , Dowsey, M. , Gunn, J. , Choong, P. , & Manski‐Nankervis, J. A. (2019). Misconceptions and the acceptance of evidence‐based nonsurgical interventions for knee osteoarthritis. A qualitative study. Clinical Orthopaedics and Related Research, 477(9), 1975–1983. 10.1097/corr.0000000000000784 31192807 PMC7000096

[msc1631-bib-0007] Butler, D. , & Moseley, G. L. (2017). Explain pain supercharged. NOI.

[msc1631-bib-0008] Caneiro, J. P. , Bunzli, S. , & O’Sullivan, P. (2021). Beliefs about the body and pain: The critical role in musculoskeletal pain management. Brazilian Journal of Physical Therapy, 25(1), 17–29. 10.1016/j.bjpt.2020.06.003 32616375 PMC7817871

[msc1631-bib-0009] Caneiro, J. P. , Sullivan, P. B. , Roos, E. M. , Smith, A. J. , Choong, P. , Dowsey, M. , Hunter, D. J. , Kemp, J. , Rodriguez, J. , Lohmander, S. , Bunzli, S. , & Barton, C. J. (2020). Three steps to changing the narrative about knee osteoarthritis care: A call to action. British Journal of Sports Medicine, 54(5). 256. 10.1136/bjsports-2019-101328 31484634

[msc1631-bib-0010] Darlow, B. , Brown, M. , Thompson, B. , Hudson, B. , Grainger, R. , McKinlay, E. , & Abbott, J. H. (2018). Living with osteoarthritis is a balancing act: An exploration of patients’ beliefs about knee pain. BMC Rheumatology, 2(1), 15. 10.1186/s41927-018-0023-x 30886966 PMC6390552

[msc1631-bib-0011] Darlow, B. , Fullen, B. M. , Dean, S. , Hurley, D. A. , Baxter, G. D. , & Dowell, A. (2012). The association between health care professional attitudes and beliefs and the attitudes and beliefs, clinical management, and outcomes of patients with low back pain: A systematic review. European Journal of Pain, 16(1), 3–17. 10.1016/j.ejpain.2011.06.006 21719329

[msc1631-bib-0012] Elo, S. , & Kyngäs, H. (2008). The qualitative content analysis process. Journal of Advanced Nursing, 62(1), 107–115. 10.1111/j.1365-2648.2007.04569.x 18352969

[msc1631-bib-0013] Fingleton, C. , Smart, K. , Moloney, N. , Fullen, B. M. , & Doody, C. (2015). Pain sensitization in people with knee osteoarthritis: A systematic review and meta‐analysis. Osteoarthritis and Cartilage, 23(7), 1043–1056. 10.1016/j.joca.2015.02.163 25749012

[msc1631-bib-0014] Fishman, S. M. , Young, H. M. , Lucas Arwood, E. , Chou, R. , Herr, K. , Murinson, B. B. , Watt‐Watson, J. , Carr, D. B. , Gordon, D. B. , Stevens, B. J. , Bakerjian, D. , Ballantyne, J. C. , Courtenay, M. , Djukic, M. , Koebner, I. J. , Mongoven, J. M. , Paice, J. A. , Prasad, R. , Singh, N. , … Strassels, S. A. (2013). Core competencies for pain management: Results of an interprofessional consensus summit. Pain Medicine, 14(7), 971–981. 10.1111/pme.12107 23577878 PMC3752937

[msc1631-bib-0015] French, S. D. , Bennell, K. L. , Nicolson, P. J. , Hodges, P. W. , Dobson, F. L. , & Hinman, R. S. (2015). What do people with knee or hip osteoarthritis need to know? An international consensus list of essential statements for osteoarthritis. Arthritis Care & Research, 67(6), 809–816. 10.1002/acr.22518 25418120

[msc1631-bib-0016] Guest, G. , Bunce, A. , & Johnson, L. (2006). How many interviews are enough? An experiment with data saturation and variability. Field Methods, 18(1), 59–82. 10.1177/1525822X05279903

[msc1631-bib-0017] Holden, M. A. , Nicholls, E. E. , Young, J. , Hay, E. M. , & Foster, N. E. (2009). UK‐based physical therapists’ attitudes and beliefs regarding exercise and knee osteoarthritis: Findings from a mixed‐methods study. Arthritis Care & Research, 61(11), 1511–1521. 10.1002/art.24829 19877105

[msc1631-bib-0018] International Pain Summit of the International Association for the Study of Pain . (2011). Declaration of Montréal: Declaration that access to pain management is a fundamental human right. Journal of Pain & Palliative Care Pharmacotherapy, 25(1), 29–31. 10.3109/15360288.2010.547560 21426215

[msc1631-bib-0019] Jackson, T. , Pope, L. , Nagasaka, T. , Fritch, A. , Iezzi, T. , & Chen, H. (2005). The impact of threatening information about pain on coping and pain tolerance. British Journal of Health Psychology, 10(3), 441–451. 10.1348/135910705X27587 16238858

[msc1631-bib-0020] Lin, I. , Wiles, L. , Waller, R. , Goucke, R. , Nagree, Y. , Gibberd, M. , & O’Sullivan, P. P. B. (2020). What does best practice care for musculoskeletal pain look like? Eleven consistent recommendations from high‐quality clinical practice guidelines: Systematic review. British Journal of Sports Medicine, 54(2), 79–86. 10.1136/bjsports-2018-099878 30826805

[msc1631-bib-0021] Louw, A. , Sluka, K. A. , Nijs, J. , Courtney, C. A. , & Zimney, K. (2020). Revisiting the provision of pain neuroscience education: An adjunct intervention for patients but a primary focus of clinician education. Journal of Orthopaedic & Sports Physical Therapy, 51(2), 57–59. 10.2519/jospt.2021.9804 33076759

[msc1631-bib-0022] MacKay, C. , Hawker, G. A. , & Jaglal, S. B. (2018). Qualitative study exploring the factors influencing physical therapy management of early knee osteoarthritis in Canada. BMJ Open, 8(11), e023457. 10.1136/bmjopen-2018-023457 PMC6278797 30498043

[msc1631-bib-0023] Marshall, B. , Cardon, P. , Poddar, A. , & Fontenot, R. (2013). Does sample size matter in qualitative research? A review of qualitative interviews in is research. Journal of Computer Information Systems, 54(1), 11–22. 10.1080/08874417.2013.11645667

[msc1631-bib-0024] Mills, K. , Hübscher, M. , O’Leary, H. , & Moloney, N. (2019). Current concepts in joint pain in knee osteoarthritis. Der Schmerz, 33(1), 22–29. 10.1007/s00482-018-0275-9 29464336

[msc1631-bib-0025] Moseley, G. L. , & Butler, D. S. (2015). Fifteen years of explaining pain: The past, present, and future. The Journal of Pain, 16(9), 807–813. 10.1016/j.jpain.2015.05.005 26051220

[msc1631-bib-0026] Nijs, J. , Paul van Wilgen, C. , Van Oosterwijck, J. , van Ittersum, M. , & Meeus, M. (2011). How to explain central sensitization to patients with ‘unexplained’ chronic musculoskeletal pain: Practice guidelines. Manual Therapy, 16(5), 413–418. 10.1016/j.math.2011.04.005 21632273

[msc1631-bib-0027] Ram, A. , Booth, J. , Thom, J. , & Jones, M. D. (2020). Exercise and education for knee osteoarthritis—what are accredited exercise physiologists providing? Musculoskeletal Care. 10.1002/msc.1477 32500962

[msc1631-bib-0028] Shipton, E. E. , Bate, F. , Garrick, R. , Steketee, C. , & Visser, E. J. (2018). Pain medicine content, teaching and assessment in medical school curricula in Australia and New Zealand. BMC Medical Education, 18(1). 10.1186/s12909-018-1204-4 PMC5948674 29751806

[msc1631-bib-0029] Somers, T. J. , Keefe, F. J. , Godiwala, N. , & Hoyler, G. H. (2009). Psychosocial factors and the pain experience of osteoarthritis patients: New findings and new directions. Current Opinion in Rheumatology, 21(5). https://journals.lww.com/co‐rheumatology/Fulltext/2009/09000/Psychosocial_factors_and_the_pain_experience_of.11.aspx 10.1097/BOR.0b013e32832ed704 19617836

[msc1631-bib-0030] Song, H. J. , Dennis, S. , Levesque, J.‐F. , & Harris, M. F. (2019). What matters to people with chronic conditions when accessing care in Australian general practice? A qualitative study of patient, carer, and provider perspectives. BMC Family Practice, 20(1), 79. 10.1186/s12875-019-0973-0 31182041 PMC6558875

[msc1631-bib-0031] Teo, P. L. , Bennell, K. L. , Lawford, B. J. , Egerton, T. , Dziedzic, K. S. , & Hinman, R. S. (2020). Physiotherapists may improve management of knee osteoarthritis through greater psychosocial focus, being proactive with advice, and offering longer‐term reviews: A qualitative study. Journal of Physiotherapy, 66(4), 256–265. 10.1016/j.jphys.2020.09.005 33036932

[msc1631-bib-0032] Thacker, M. A. , & Moseley, G. L. (2012). First‐person neuroscience and the understanding of pain. Medical Journal of Australia, 196(6), 410–411. 10.5694/mja12.10468 22471546

[msc1631-bib-0033] The Royal Australian College of General Practitioners . (2018). Guideline for the management of knee and hip osteoarthritis (2nd ed.).

[msc1631-bib-0034] Tong, A. , Sainsbury, P. , & Craig, J. (2007). Consolidated criteria for reporting qualitative research (COREQ): A 32‐item checklist for interviews and focus groups. International Journal for Quality in Health Care, 19(6), 349–357. 10.1093/intqhc/mzm042 17872937

[msc1631-bib-0035] Tracy, L. M. (2017). Psychosocial factors and their influence on the experience of pain. Pain Reports, 2(4). 10.1097/PR9.0000000000000602 PMC5741357 29392217

[msc1631-bib-0036] Vasileiou, K. , Barnett, J. , Thorpe, S. , & Young, T. (2018). Characterising and justifying sample size sufficiency in interview‐based studies: Systematic analysis of qualitative health research over a 15‐year period. BMC Medical Research Methodology, 18(1), 148. 10.1186/s12874-018-0594-7 30463515 PMC6249736

[msc1631-bib-0037] Williams, A. C. d. C. , Fisher, E. , Hearn, L. , & Eccleston, C. (2020). Psychological therapies for the management of chronic pain (excluding headache) in adults. Cochrane Database of Systematic Reviews, (8). 10.1002/14651858.CD007407.pub4 PMC7437545 32794606

