# Supplementary material for: Exercise physiologists use of pain neuroscience education for treating knee osteoarthritis: A qualitative interview study
Source: Musculoskeletal Care. 2022 Mar 17;20(4):821–30. doi: 10.1002/msc.1631 (PMC10078781; doi:10.1002/msc.1631)
Supplement: Supplementary file 1 — Supporting Information S1 [file MSC-20-821-s002.docx]

Supplementary Material 1

**Patient**: ♀ 54 yrs        Ht: 175 cm       Wt: 79kg       Work: Aged health care nurse 25 h/week

**Diagnosis**:   Right knee OA diagnosed 2014   Other: No other known comorbidities

**Previous investigations**: MRI and arthroscopy >20 yrs for meniscal injury. No recent scans

**History**: Previous treatment: Physiotherapy, 0steopathy previous 5 yrs involving electro modalities, heat therapy, massage and exercise

Symptoms: Morning stiffness worse on first waking (5/10) gradually reduces. Frequent crepitis and joint pain/discomfort. Patient report "clicks and grinds like a rusty hinge". Intermittent infrapatellar and joint space swelling. Constant pain 1-3/10 occasionally increasing to 7/10. Pain free at rest. No red flags

Aggravating factors: Walking > 20-30 min, squatting, kneeling/crouching, stair climbing particularly descending, stress

Relieving factors: Rest, NSAIDs, Panadol osteo, massage

Screening questionnaires (attached): WOMAC 53%;pain Self-efficacy 20/60

Thoughts, beliefs, coping: Physio diagnosed and explained knee OA. Pt believes her knee is getting worse and requires MRI. Pt report "things that didn't use to hurt are painful and my hip, L knee and even other parts of my body are starting to hurt now". Frustrated and anxious about possibility of longer-term disability. Believes she should stop activity with ↑pain because Pt "doesn't want to cause further damage".  Limits her walking and avoid stairs, kneeling and crouching and demanding physical activities (e.g. mowing), uses medication as required.

Physical examination: **Observation**: No swelling comparing R & L side **ROM**: Knee: flexion 120^0^; extension 0^0^; Stiffness and end range discomfort during ROM testing **Manual muscle testing**: Gluteal weakness worse on R side; R knee flexor and extensor muscle weakness worse on right side. **Function**: Walking tolerance 20-30 min; shortened gait, obvious limp; self-paced sit to stand test 8 reps ↑pain; unable to squat; required handrail stairclimbing avoided stepping down on R leg ↑pain. **Behaviours:** Vigilant of symptoms during activity, self-limits activity that ↑pain, pain behaviours (grimacing, compensating with L leg)
