# Supplementary material for: Exercise physiologists use of pain neuroscience education for treating knee osteoarthritis: A qualitative interview study
Source: Musculoskeletal Care. 2022 Mar 17;20(4):821–30. doi: 10.1002/msc.1631 (PMC10078781; doi:10.1002/msc.1631)
Supplement: Supplementary file 2 — Supporting Information S2 [file MSC-20-821-s001.docx]

| Questions |  |
| --- | --- |
| 1. How would pain education be helpful for this patient? | |
| 1. The patient believes that her degenerative knee changes are the cause of her pain and impaired function. What pain education could you draw on to respond to this? | |
| 1. The patient strongly believes that she requires an MRI. Do you agree? Yes or no | |
| 1. The patient believes pain is an indication that she could be causing further damage and to cease activity. What pain education would you provide? | |
| 1. What education would you provide about pain during exercise? | |
| 1. Metaphors like (exercise is medicine) and analogies can be very powerful pain education tools. Can you tell me a few metaphors/analogies that might be used for this patient? | |
| 1. Patient self-reports "my knee clicks and grinds like a rusty hinge". What pain education might you provide? | |
| 1. Pt report "things that didn't use to hurt are painful and my hip, L knee and even other parts of my body are starting to hurt now". What pain education would you provide? | |
| 1. Pt reports relieving factors as rest, NSAIDs, Panadol osteo, massage. What pain education would align with developing better coping and self-management | |
| 1. Is there other pain education that we haven't touched on that you consider might be important for this knee OA Pt. | |
| 1. Can you briefly tell me what pain science education you have received or engaged in? | |

Supplementary Material 2: Semi-structured interview questions based on knee osteoarthritis vignette
